# Oxidation-responsive, settable bone substitute composites for regenerating critically-sized bone defects†

**DOI:** 10.1039/d4bm01345j

**Published:** 2025-02-21

**Authors:** Reinaldo L. Dos Santos, Ardeena Ahmed, Brooke E. Hunn, Adolphus E. Addison, Dylan W. Marques, Karina A. Bruce, John R. Martin

**Affiliations:** a Biomedical Engineering, University of Cincinnati Cincinnati Ohio USA marti7j3@ucmail.uc.edu

## Abstract

Critically-sized bone defects that cannot spontaneously heal on their own remain a significant problem in the clinic. Synthetic polymeric implants are promising therapies for improving bone healing as they are highly tunable and avoid the potential complications associated with autologous bone grafts. However, biostable implants such as poly(methyl methacrylate) (PMMA) suffer from numerous shortcomings including negligible biodegradability and limited osseointegration with bone. Hydrolytically-degradable polymeric implants such as poly(caprolactone) (PCL) or poly(lactic-*co*-glycolic acid) (PLGA) have shown promise facilitating bone growth before being resorbed, but matching the degradation rate of these polyesters with the rate of bone regeneration continues to be an engineering challenge. To address these limitations with current synthetic bone implant materials, cell-degradable polymer/hydroxyapatite composites were developed as *in situ*-curing bone substitutes. The polymeric component was formulated from a thioketal (TK) dithiol linker and a tri-functional epoxy to facilitate rapid crosslinking upon deployment. To enable biologically-responsive implant resorption, the TK unit is specifically cleaved by cell-produced reactive oxygen species (ROS). TK bone substitutes possessed tunable curing and mechanical properties, were selectively degraded in dose-dependent concentrations of ROS, were non-cytotoxic, and demonstrated significantly greater bone regeneration capacity than PMMA in a critically-sized rat skull defect model. These combined results highlight the therapeutic potential of cell-degradable bone void fillers compared against conventional polymeric bone implants.

## Abstract 2

Traditionally used bone substitutes do not yield desired bone regeneration outcomes in critically-sized bone defects. Oxidation sensitive bone substitutes are an alternative that produce improved regeneration in large-scale bone defects.

## Graphical abstract

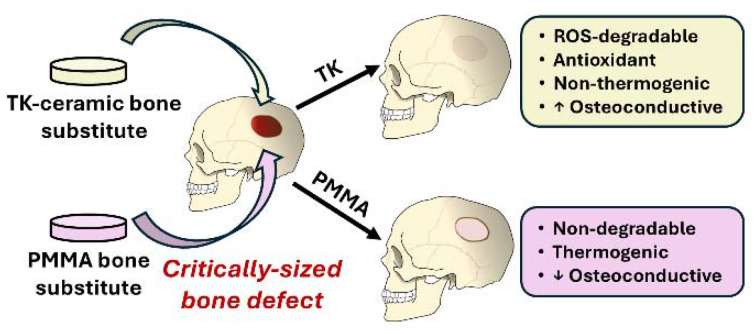

## 1. Introduction

Critically-sized bone defects that cannot spontaneously heal on their own remain a significant problem in the clinic.^1–5^ The incidence of these medical complications arises from several origins, including osteotomy surgeries addressing infection or cancer of the bone, trauma, and congenital disorders.^2,3,6^ The gold-standard treatment for these large-scale bone injuries are autologous bone grafts where intact bone tissues from the patient's hip or fibula are extracted, re-shaped, and implanted into a defect site.^7^ Off-the-shelf synthetic bone substitution materials^8,9^ are particularly promising as they avoid the additional surgeries and potential complications inherent with autologous bone grafting. A common bone substitute utilized in the clinic, polymethyl methacrylate (PMMA), has been implemented widely to stabilize injured bone tissue because of its ability to harden *in situ* upon deployment.^8,10^ When employed properly, PMMA cements possess negligible cellular toxicity, achieve high weight bearing levels, and can mediate some limited osseointegration between total joint replacement implants and bone.^11^The high strength of PMMA cements allows for its use stabilizing load-bearing bone,^12^ as an implant fixator in total joint replacement surgeries,^13^ and as dental fillings.^14^ However, PMMA suffers from innate shortcomings as a bone substitute due to its exothermicity upon setting, a lack of biodegradability after implantation, limited osteointegration over time, and potentially toxic unpolymerized constituents from monomer leakage.^13–16^ Therefore, developing synthetic bone substitutes that can improve bone regeneration over current clinically-approved biomaterials remains a top priority in orthopedic research.

To address issues observed with using PMMA in bone augmentation, many biodegradable polymer–ceramic composites have been investigated in recent years.^17^ These approaches rely on implanting biodegradable materials into an injury site where they serve as scaffolds for guiding tissue repair before degrading into non-toxic byproducts that can be cleared from the body. The most widely explored synthetic biodegradable materials for bone regeneration include hydrolytically-degradable polyesters such as poly(caprolactone) (PCL), poly(lactic acid) (PLA), and poly(lactic-*co*-glycolic acid) (PLGA). Though these polymers are generally considered non-toxic,^18,19^ they do not inherently induce new bone growth and have often been combined with biologically-relevant ceramics such as hydroxyapatite, tri-calcium phosphate, or bioglass to enhance both their mechanical properties and osteoconductivity.^20–23^ Pure ceramic implants suffer from poor mechanical properties,^24,25^ making polymer/ceramic composites particularly attractive as synthetic bone substitutes since they can combine the favorable biological attributes of ceramics with the increased material toughness imparted from a crosslinked polymer network.^17^ Clinical utilization of these bone substitute biomaterials are ongoing with some success;^10,26,27^ however, matching the rate of polyester degradation with bone regeneration continues to be an ongoing engineering challenge.^18^ The resulting mismatch can result in incomplete bone healing from prematurely degraded scaffolds,^28^ or conversely, tissue fibrosis from implants that degrade too slowly.^29^ To this end, environmentally-responsive “smart” polymers that are specifically degraded by cell-generated signals during bone remodeling have recently emerged. Most notably, these include implants which specifically respond to bone-specific enzymes^30–32^ or cell-generated reactive oxygen species (ROS).^33,34^

Bone remodeling after initial injury involves a plethora of cell types and signaling cascades which ultimately lead to bone regrowth.^35,36^ One of the key components of this process are oxidative free radicals which feature heavily in redox signaling during the initial stages of inflammation. While ROS plays a key role in cellular differentiation leading to bone remodeling, extended bouts of oxidative stress are a hallmark of disease pathogenesis and can negatively influence bone regeneration outcomes.^33,37,38^ This has led to the development of antioxidant-loaded implants that improve bone healing,^39,40^ and more recently, tissue engineering scaffolds that are selectively degraded by ROS.^41–43^

Oxidation-responsive polymer systems are a developing class of synthetic biomaterials with exciting medical applications.^33,44^ Currently, several ROS-activatable chemistries exist but generally fall into one of two classes depending on whether they feature an oxidation-induced phase transition or oxidation-induced covalent degradation. The thioketal (TK) moiety has been a particular group of interest for tissue engineered scaffolds due to its relatively simple formation through condensation polymerization,^45^ selective covalent cleavage of TK polymer chains by ROS molecules,^46^ and ability to completely degrade and clear from the body.^47^ The creation of TK-containing polyurethane scaffolds has been the subject of recent efforts to address inherent issues with other synthetic bone substitutes.^43,48–51^ Though promising, these ROS-degradable polyurethanes are limited by the multi-step synthesis required to generate TK diol precursors^43^ and the unstable, highly-reactive, and potentially toxic poly-isocyanate crosslinkers used to generate the final materials.^52^ Furthermore, polyurethanes are notoriously exothermic during their formation^53,54^ which can limit their utility as *in situ*-hardening bone substitutes.

This current work describes the creation of a TK/epoxy/hydroxyapatite composite as a bone substitute material. A dithiol TK was synthesized through a one-pot synthesis, mixed with osteogenic hydroxyapatite crystals, and crosslinked *via* a thiol-epoxy ring opening polymerization to create TK-ceramic scaffolds. The curing time, mechanical properties, oxidative degradation, antioxidant capacity, and cell viability of these materials were assessed *in vitro*. Additionally, TK-ceramic composites were implanted in a rat calvarial defect model to screen biocompatibility and regeneration capacity *in vivo*. Encouragingly, these ROS-degradable composite materials promoted significant bone formation while addressing many of the limitations reported with current synthetic bone substitutes.

## 2. Methods

### 2.1. Materials

All chemicals and reagents were purchased from Fisher Scientific (Waltham, MA), except for the following. 2,2 – Diphenyl-1-picrylhydrazyl (DPPH), 100 μm hydroxyapatite (HaP) (product # 289396), and cobalt chloride (CoCl_2_) were acquired from MilliporeSigma (St Louis, MO). Modified essential medium α (αMEM) with no ascorbic acid, penicillin–streptomycin-glutamine (100×), and fetal bovine serum (FBS) were acquired from Gibco (Waltham, MA). The CellTiter-Glo assay kit was acquired from Promega (Madison, WI). Buprenorphine Extended Release (Bup-ER) at 0.5 mg mL^−1^ and Meloxicam Extended Release (Melox-ER) at 2 mg mL^−1^ were obtained from Wedgewood Pharmacy. Teets ‘cold cure’ PMMA dental cement solvent and powder were acquired from A-M Systems (Sequim, WA). An alkaline phosphatase assay kit (colorimetric) was obtained from Abcam (United Kingdom). All materials were used as received unless otherwise specified.

### 2.2. SCTK synthesis

Methods for preparation of the SCTK dithiol were adopted and optimized from Martin *et al.*^41^ Briefly, a stir-bar and *p*-toluenesulfonic acid monohydrate (5.6823 g) were added to a 1000 mL tri-neck round bottom flask adapted with a 250 mL addition funnel and stopcock. The vessel was placed under high vacuum for 20 minutes and purged with nitrogen. The round bottom flask and addition funnel were then respectively charged with 300 mL and 100 mL of anhydrous acetonitrile. In the round bottom flask compartment, 180 mL (1.1 mol) of 3,6-dioxa 1,8-octanedithiol (DOT) was added. The addition funnel was then charged with 67.4 mL (0.55 mol) of 2,2-dimethoxypropane (DMP) in a 1 : 2 molar eq. to DOT. A molar excess of dithiol monomer was used to generate short-chain TK dimers (SCTK) with minimized molecular weight in the final product. The reaction vessels were then degassed with nitrogen in both the round bottom flask and addition funnel for 10 minutes each. Upon completion of degassing, the DMP-acetonitrile solution was added drop-wise into the round bottom flask with continuous stirring for 5 hours. The final solution was then transferred to a 1000 mL single neck round bottom flask to remove the acetonitrile *via* rotary-evaporation. The crude SCTK product was then purified *via* three rounds of precipitation in chilled volumes of 70 : 30 isopropanol and water. The obtained dithiol product was then analyzed *via* GPC using poly(ethylene glycol) (PEG) dithiol molecules as standards (122 Da–3400 Da) to estimate molecular weight. The GPC analysis was done using an Ultimate 3000 system (Thermo Fisher Scientific, Waters, MA) with a dimethylformamide (DMF) mobile phase and a Styragel HR 1 DMF column (Waters Corporation, Milford, MA). To confirm successful polymerization and purification, the product was dissolved in deuterated chloroform (CDCl_3_) and analyzed with ^1^H nuclear magnetic resonance spectroscopy (NMR, Bruker AV 400 spectrometer). ^1^H NMR chemical shifts are reported as *δ* values in ppm relative to CDCl_3_ (*δ* = 7.28). Peak multiplicity is reported as: s (singlet), d (doublet), t (triplet), and m (multiplet). Protons representative to each peak are assigned as nH, where n represents the number of hydrogens corresponding to specific peaks based on peak integration. ^1^H NMR: *δ* = 3.65–3.54 (d, 8H), *δ* = 2.80 (t, 2H), *δ* = 2.67 (t, 2H), *δ* = 1.62 (s, 1H), *δ* = 1.60 (s, 6H).

### 2.3. Formation of TK-ceramic composite scaffolds and PMMA control materials

SCTK and trimethylolpropane triglycidyl ether (tri-epoxy) were weighed in separate 2 mL micro-centrifuge tubes in amounts that matched molar concentrations of thiol groups with epoxy groups from the two respective components. Choline (47–50% in water) at 5.3 mass % (mass of catalyst/mass of polymer in the scaffold) was added to the container with the tri-epoxy and vortex mixed for 1 minute. HaP was weighed out in batch-specific amounts corresponding to the total ceramic quantity desired in the final scaffold formulations. HaP was deposited into a glass crystallization dish and formed into a crater for thorough incorporation of liquid components. SCTK was first aspirated and deposited into the HaP before thoroughly mixing with a spatula until a stiff tacky consistency was achieved. The tri-epoxy/choline mixture was then aspirated and deposited onto the HaP-SCTK slurry and mixed vigorously with a spatula until a runny white mixture was achieved. The pre-cured TK-epoxy-ceramic slurry was then transferred into soft 6 mm-diameter low density polyethylene (LDPE) tubes (Fisher Scientific catalog #033381A) which functioned as cylindrical molds. The samples were allowed to cure at room temperature for 1–7 days. PMMA scaffolds were formed by incorporating 3 parts Teets “cold cure” powder and 1 part solvent by volume per the manufacturers recommendations. Powder and liquid were mixed thoroughly and allowed to stand for 5 minutes. The mixture was then pressed into molds and allowed to cure for 24 h. Lastly, to measure any thermogenicity of TK-ceramic composites and PMMA scaffolds, components were added to a glass crystallization disk and initially homogenized thoroughly to initiate curing. A Kizen LaserPro LP300 infrared thermometer was used to determine temperature of curing TK and PMMA materials every 30 s after homogenization. Each test was conducted until a tack-free TK scaffold was achieved which lasted about 15 minutes.

### 2.4. Characterization of TK scaffold properties

Tack-free time of curing TK-ceramic scaffolds was measured in triplicate following protocols listed in ASTM C679. Briefly, scaffold components were mixed and homogenized and a timer started. Every 5–10 s the curing mixture was probed with a spatula and analyzed for adherence to its surface. The timer was stopped once all material did not adhere to the spatula to determine the recorded tack-free time. An antioxidant capacity assay was done using 1,1-diphenyl-2-picrylhydrazil (DPPH).^55,56^ Desired timepoints for this experiment were 1, 2, 3, 6, 8, 16, and 24 h of incubation with *n* = 3 replicates for each timepoint. TK-ceramic and PMMA scaffolds were sectioned into 10 mg portions (40, 50, or 60% mass fraction) and were added into 4 mL glass vials and assigned a replicate and timepoint. DPPH solution was prepared by dissolving 7.905 g of DPPH into 100 mL of 80 : 20 v/v% of 200 proof ethanol and deionized (DI) water before wrapping the bottle in aluminum foil to prevent light contamination. In light-free conditions, 2 mL of DPPH solution was added into every scaffold-containing vial which were then placed in a closed carboard box and incubated at 37 °C on a shaker table for the duration of the study. Upon completion of a timepoint, 100 μL of solution was added to a 96-well plate and absorbance was measured on a Tecan MPlex microplate reader at 517 nm. Absorbance of the material-treated DPPH solutions (*A*_Sample_) was normalized to absorbance of a non-treated (no scaffold) DPPH solution aliquot (*A*_Control_). % DPPH was determined using the following equation:
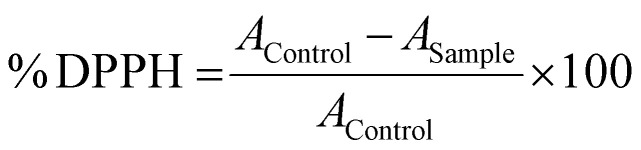


Sol-fraction experiments were also conducted to determine the unreacted components in the crosslinked materials. TK scaffolds made with 50% mass fraction HaP or without HaP were fabricated as described above, cured for 24 h, weighed, and then incubated in reagent alcohol for 48 h. Non-HaP containing scaffolds were desired to assess potential HaP leaching from formed scaffolds. Reagent alcohol was used due to its ability to dissolve any unreacted polymeric components within scaffolds. Upon completion of incubation, samples were removed and air dried for 24 h and subsequently lyophilized for another 24 h before being weighed.

### 2.5. Scanning electron microscopy

TK scaffolds (50% HaP mass fraction) were sectioned and sputter coated with gold particles for 5 seconds in preparation for imaging. Samples were imaged using a ThermoScientific Apreo C (Waltham, MA) scanning electron microscope (SEM) to elucidate material microstructure.

### 2.6. TK scaffold mechanical properties

TK-ceramic scaffolds with varying ceramic loading (40, 50, 60% mass fraction of HaP) were formulated as described above into 6 mm-diameter cylinders, cured for up to 14 days at room temperature, and prepared corresponding to desired timepoints for testing. A TestResources universal test machine (model 100R6) with 112 lbf force transducer (model SMT2-112-294) was used to assess the compressive properties of TK scaffolds. Before testing, the 6 mm cylindrical samples had their ends flattened using sandpaper and measured with calipers to determine exact diameters and gauge lengths. Samples were loaded onto compression plates and a pre-load of 2 N was established. Scaffolds were then compressed at a rate of 1.3 mm min^−1^ per protocols outlined in ASTM D6641. Force-displacement data were then converted to stress–strain curves based on the initial geometries of the respective tested samples. The elastic modulus was determined from the linear region of the stress–strain curve and calculated using the point slope formula. Yield strain and yield stress were measured at the coincidence between the stress–strain curve and a 2% offset line in the plastic deformation region. Toughness was measured as the area under the stress–strain curve until ultimate failure using the trapezoid method. Lastly, TK scaffold wet properties were analyzed by forming scaffolds in molds as previously described, removing samples from their molds after 24 h of initial hardening, and then incubating samples in phosphate buffered saline (PBS) at 37 °C for 14 days. On day 14 similar methods for dry sample compression testing were employed for assessing compressive properties of the wet specimens.

### 2.7. *In vitro* TK scaffold degradation

To assess TK scaffold degradation, samples with 50% HaP were formulated, cured for 5 days at room temperature, cut into ∼20 mg samples, and then soaked in reagent alcohol for 24 h to remove any potential unpolymerized materials. Upon drying completely for 24 h, samples were weighed before placing in 2 mL microcentrifuge tubes and assigned a timepoint with *n* = 3 replicates. Long-term aqueous stability was assessed by completely submerging scaffold samples in 1 mL PBS, incubating on a shaker table at 37 °C, and measuring sample mass loss after the desired timepoint was achieved. Buffer media was replaced every 3–4 days. Upon removal at the desired timepoint, samples were rinsed in deionized (DI) water thoroughly and dried for 48 h before recording their final mass. Oxidative degradation of TK-ceramic samples was assessed using similar methods but instead employing a protocol that simulates *in vivo* oxidative degradation over an accelerated time scale.^57^ Oxidative media was prepared using DI water with 20% hydrogen peroxide (H_2_O_2_) and 0.1 M cobalt chloride (CoCl_3_); cobalt ions react with the H_2_O_2_ to stimulate the formation of highly reactive hydroxyl radicals. Samples were washed in reagent alcohol, dried for 24 h, incubated on a shaker table at 37 °C, and assigned a timepoint in triplicate. Upon timepoint completion, samples were removed and washed thoroughly in DI water and dried for 48 h before recording their final mass. Oxidative media was replaced every 3–4 days. To determine rate changes in degradation as a function of oxidative media concentration, 20% H_2_O_2_ media was diluted tenfold to achieve 2% H_2_O_2_ in 0.01 M CoCl_3_. Methods outlining mass loss assessment in oxidative media were similarly followed with samples incubated in 2% H_2_O_2_. Lastly, the swelling ratios of TK scaffolds were determined after incubation in PBS or 20% H_2_O_2_ for 7 days. Lead candidate 50% HaP TK scaffolds were formed and allowed to cure over 24 h. Scaffolds were removed from their molds, sectioned into approximately 300 mg sections, washed in reagent alcohol, and lyophilized for 24 h. The dry weight (*W*_Dry_) of each sample was recorded before incubating them in PBS for another 24 h and collecting the samples’ wet weight (*W*_Wet_) values. After this initial incubation, *n* = 3 samples were placed in either PBS or 20% H_2_O_2_ for an additional 7 days before collecting their *W*_Wet_ and *W*_Dry_ values as described above. Each sample's swelling ratio was calculated using the following equation:
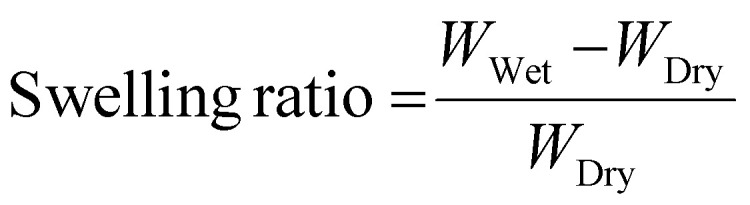


### 2.8. Cytotoxicity of choline catalyst and TK scaffolds

All cell culture experiments were carried out using MC3T3-E1 pre-osteoblast cells (American Type Culture Collection, ATCC) incubated under sterile conditions at 37 °C with 5% CO_2_. Media for these cells used a base αMEM (no ascorbic acid) supplemented with 10% FBS and 1% penicillin/streptomycin. To determine acute cellular toxicity of the choline catalyst (received from the manufacturer as a solution with 47–50% water), this reagent was added to full cell media in incrementally increasing concentrations of 0.5, 2.5, 5, and 10 mg mL^−1^, incubated at 37 °C for 24 h, and sterile filtered afterwards. MC3T3-E1 cells were seeded in a 96-well plate (1 × 10^4^ cells per well) for 24 h, treated with varying concentrations of choline-doped media for 24 h, and then measured for number of viable cells using a CellTiter-Glo assay as previously described.^58,59^*In vitro* cytotoxicity of TK-ceramic scaffolds (50% HaP) was assessed using an elution assay following protocols outlined in ISO 10993-5. TK scaffold samples were fabricated as described above and used directly from the mold without alteration. Scaffolds were segmented into roughly 300 mg individual samples, autoclaved, incubated in approximately 2 mL culture media for 24 h (150 mg scaffold per mL media), and then sterile filtered. MC3T3-E1 cells were seeded onto a 96-well plate at an initial density of 1 × 10^4^ cells per well and allowed to adhere for 24 h. Cells were then treated for 24 h with serially diluted conditioned media from the TK-ceramic scaffolds using *n* = 3 seeded wells per treatment. Full unmodified media was used as a control and diluent. A CellTiter-Glo assay was used to assess the number of viable cells per treatment at the final timepoint.

For experiments where cells were seeded directly onto scaffolds, samples of TK-HaP, PMMA, and “pure TK” (the same TK-HaP scaffold without the HaP) were cast into 8 mm diameter plastic molds and allowed to cure for 24 h. Scaffolds were then removed from their molds and sectioned into 8 × 1 mm discs, washed in reagent alcohol, and autoclaved. Scaffold samples were then placed into wells of a 24-well plate before being seeded with MC3T3-E1 cells with an initial density of 5 × 10^4^ cells per well. A CellTiter-Glo assay was used to assess the number of viable cells per treatment at 24 and 72 h after initial seeding (*n* = 3 seeded scaffolds per formulation at each time point).

### 2.9. *In vitro* cellular osteogenesis mediated by TK-ceramic scaffolds

MC3T3-E1 cells were incubated with varying scaffold formulations and assessed for their production of alkaline phosphatase (ALP), a common marker of cellular osteogenesis.^60^ For measurement of ALP activity, 8 × 1 mm discs of TK-HaP, PMMA, and pure TK scaffolds were produced and sterilized using similar methods as described above. MC3T3-E1 cells were initially seeded onto a 24-well plate at a seeding density of 5 × 10^4^ cells per well, allowed to adhere for 24 h, and then supplied with a respective scaffold sample added to the well directly on top of the cells (*n* = 3 treatment scaffolds per formulation). An osteogenic differentiation medium (αMEM medium supplemented with 100 μg mL^−1^ ascorbic acid and 10 mM β-glycerophosphate^61^) was then administered to the cell/scaffold wells to induce cellular osteogenesis over 7 days. Before beginning the ALP activity assay, media were aspirated and cells were washed with PBS, trypsinized, and placed into 2 mL microcentrifuge tubes. The cells were then lysed in cold PBS by vigorous vortex mixing and the lysates were processed to measure ALP activity according to manufacturers instructions (Abcam). Briefly, the lysates were resuspended in 500 μL assay buffer and centrifuged for 15 minutes at 16,000 rpm. The supernatant samples were aspirated (400 μL) and transferred to a separate 2 mL microcentrifuge tube where they were kept on ice for the remainder of the protocol.

Supernatant samples were transferred into the wells of a 96-well plate at a volume of 80 μL before adding 50 μL of *p*-nitrophenyl phosphate (pNPP) solution to each well. After 60 minutes of incubation in the dark, 20 μL of stop solution was added to each sample well and absorbance at 405 nm was recorded using a Tecan MPlex microplate reader. Absorbance of the background was also taken and subtracted from the sample signal. Lastly, *p*-nitrophenol (pNP) concentration was determined using a standard curve with naïve ALP enzyme. ALP activity was calculated using the following equation:ALP activity (U mL^−1^) = (*A*/*V*)/*T* where *A* is the amount of pNP generated in samples determined from the standard curve (μmol), *V* is the volume of sample added into the well (mL), and *T* is the reaction time in minutes. A Bradford assay was utilized in conjunction with the ALP activity results to normalize signal to the number of cells within each well. Briefly, 250 μL of Coomassie reagent (Bioworld, Irving TX) was added to 5 μL of supernatant in 1.5 mL centrifuge tubes and allowed to incubate for 10 minutes at room temperature. Samples were then added to a 96-well plate at 100 μL and their absorbance was taken at 595 nm using a Tecan MPlex microplate reader. Sample protein amounts were calibrated using a standard curve prepared with bovine serum albumin protein.

### 2.10. *In vivo* implantation of TK scaffolds

All animal studies were performed in accordance with the Guidelines for Care and Use of Laboratory Animals and were approved by the University of Cincinnati Institutional Animal Care and Use Committee (IACUC) as documented in IACUC protocol # 22-04-22-01. All facilities are fully accredited by the Animal Welfare Assurance (AAALAC, # A-3295-01) and fully comply with the Public Health Service Policy on the Humane Care and Use of Laboratory Animals (PHS Policy) and the United States Department of Agriculture (USDA) Animal Welfare Act and Regulations (AWA and AWR). Calvarial defect surgeries were conducted following methods from Spicer *et al.*^62^ TK scaffolds (50% HaP mass fraction) were prepared as described above and deposited in a custom-made 8 mm cylindrical aluminum mold to cure over 48 h at room temperature. Cylindrical cements were sectioned transversely at a thickness of 0.8–1 mm using a High-Tech Diamond low speed saw (part #22-227) (Westmont, IL) to generate the final implants. Similarly, PMMA was prepared using Teets cold cure dental cement (Sequim, WA) in the previously described custom mold and sectioned transversely at the same thickness of 0.8–1 mm. TK and PMMA samples were then autoclaved for sterilization in preparation for implantation. Methods from Spicer *et al.*^62^ describing evaluation of bone regeneration in critically-sized rat calvarial defects were followed. Briefly, *n* = 8 male Sprague-Dawley rats at a weight of 300–325 g were obtained from Charles River (Wilmington, MA). Rodents were placed in an induction chamber with 4% (vol/vol) isoflurane and oxygen and anesthetized for 2 min. Anesthesia was maintained using a rodent nose cone under constant 2% (vol/vol) isoflurane and oxygen for the duration of the surgical procedure. Pre-operation, buprenorphine-ER and meloxicam-ER were delivered subcutaneously as analgesics at doses of 1.2 and 2.0 mg kg^−1^, respectively. Upon anesthesia administration and confirmation of animal knockout *via* toe pinch, rat skulls were shaved from the bridge of the snout to the caudal end of the calvarium using electric clippers and the skin prepared using betadine and 70% ethanol. The rat was then transferred to a 37 °C heating pad where a 1.5 mm scalpel incision was made through the skin and periosteum along the calvarium. The periosteum was carefully conserved by pushing laterally and securing with hemostats. A Nobelpharma DEA031 drill controller system (Tokyo, Japan) with KaVo intramatic 7c surgical drill (Biberach, Germany) and 8 mm trephine operated at 1500 rpm were used to initially score the calvarium along the sagittal suture. Trephination was continued carefully with a constant saline drip of 1 drop every 2 seconds until initial trephine penetration through the skull was achieved. Flat end forceps were used to pull up the bone flap from the surrounding skull before separating the underlying blood vessels from the created bone flap. Rongeurs were used to further trim the defect to conform to the desired 8 mm implant size. Implant samples were then carefully inserted into the defect, with *n* = 4 animals receiving TK-ceramic implants and *n* = 4 animals receiving PMMA implants. The periosteum and skin were sutured using VeterSut 4-0 polyglactin sutures along the incision length using interrupted stitches. Rats were administered 5 mL kg^−1^ sterile saline upon completion of the procedure and administered an additional dose of buprenorphine-ER after 72 h. Rats were housed for 8 weeks post operation before being euthanized by CO_2_ inhalation and secondary thoracic puncture.

### 2.11. Microcomputed tomography

Microcomputed tomography (microCT) imaging of rodents’ skulls was performed using a Siemens Inveon PET/SPECT/CT scanner (Siemens Medical Solutions, Malvern, PA, USA). MicroCT imaging of live anesthetized animals was conducted at 4 weeks after initial surgery, while CT imaging of freshly euthanized animals was conducted at week 8 post-surgery. The cone-beam CT parameters were as follows: 360° rotation, 720 projections, 1100 ms exposure time, 80 kVp voltage, 500 μA current, and effective pixel size 44.21 μm. Acquisitions were reconstructed using a Feldkamp algorithm with slight noise reduction, a Shepp-Logan filter, and a beam-hardening correction for rats for a 3D matrix size 1024 × 1024 × 1536 using manufacturer-provided software. A protocol-specific Hounsfield Unit (HU) calibration factor was applied. Bone analysis was conducted using Microview software (Parallax Innovations) where bone volume and tissue mineral content were calculated from an 8 mm diameter and 1 mm thick ROI at the site of implantation. Due to the calcium phosphate content of the HaP in the implanted TK scaffolds, bone and scaffold mineral phases were very similar and difficult to separate during bone analysis. To establish baseline values for TK-ceramic samples’ bone volume and bone mineral density, an 8 mm defect was first created in the skull of a fresh rat cadaver as described above and then implanted with a newly-made TK ceramic scaffold. MicroCT was performed on the day 0 defect/TK implant and analyzed at various thresholds to determine a baseline value for bone volume and bone mineral density for TK scaffolds. When analyzing microCT data from TK scaffolds implanted for 4 or 8 weeks, the day 0 implant's baseline values for BV and BMC at matched thresholds were subtracted from the measured data to isolate BV and BMC signal generated by newly formed bone. Further details outlining bone analysis thresholding choices are explained in the supplemental document.

### 2.12. Histology

Following postmortem CT imaging, rat skull tissues were harvested to obtain the top of the calvarium including the implant and surrounding bone. Each sample was placed in its own vial of 10% (vol/vol) formalin for fixation lasting a week. Samples were then removed and decalcified using 10% formic acid in PBS over 5 days. Following bone decalcification, samples were bisected along the sagittal suture for embedding. Because PMMA samples were too hard for conventional histological sectioning, these implants were removed from decalcified tissues before embedding. Day 0 rat calvarial defects with implanted TK scaffolds were created in cadaver skull samples as described above and also submitted for histology following this fixation, decalcification, and embedding procedure. Samples were placed cut side down during embedding and serially sliced at 5 μm sections. Each section was stained with hematoxylin and eosin (H&E) for tissue visualization. Though TK implants were not removed from the tissues before embedding like the PMMA samples, the sequential solvent washes during the staining process removed most of the residual TK material from the final sections. All stained samples were imaged using an Olympus IX83 microscope (Tokyo, Japan) and analyzed using ImageJ.

### 2.13. Statistical analysis

The data presented here are reported as mean and standard deviation of the mean. Statistical analyses for comparing two groups was performed using a Student's *t*-test. Statistical analyses for multiple group comparisons were preformed using one way analysis of variance (ANOVA) including a Tukey's *post-hoc* test. Statistical analyses for matched implant groups compared at different time points were performed using a repeated measures *t*-test. *P*-Values less than 0.05 were considered to be statistically significant.

## 3. Results and discussion

### 3.1. Formation of settable TK polymer/ceramic scaffolds

In this work, four components were combined to generate oxidation-sensitive bone implants: an ROS-degradable TK linker to confer cell-specific material degradation, a tri-functional epoxy crosslinker that could covalently bond with the TK's thiol groups to form a fast-hardening polymer network, a low dose of choline catalyst, and a hydroxyapatite ceramic that could be homogenously mixed with the reactive polymer mixture to improve the final materials’ mechanical properties and osteointegration. First, a TK oligomer with homobifunctional thiol end groups was synthesized from 2,2 dimethoxypropane (DMP) and 3,6-dioxa-1,8-octanetihiol (DOT) as adapted from previous guidelines.^41^ Most previously reported examples of TKs synthesized from dithiol monomers have generated polymers with chain lengths near 1000 g mol^−1^.^41–43^ Using similar synthesis methods, initial efforts here also generated TK dithiol polymers with number average molecular weight (*M*_n_) values near 1000 g mol^−1^ as calculated from gel permeation chromatography (GPC). However, ceramic-loaded scaffolds generated from these TK polymers featured poor mechanical properties with modulus values of less than 1 MPa (data not shown).

**Fig. 1:**
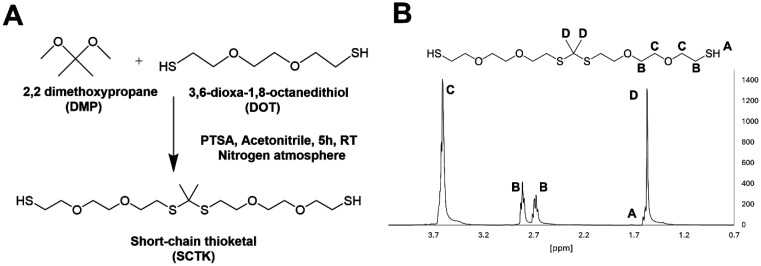
(A) Chemical synthesis scheme for SCTK dithiol, the cell-responsive precursor used to generate bone substitute materials when combined with a tri-epoxy crosslinker and hydroxyapatite. (B) ^1^H NMR of SCTK indicating successful TK product formation.

Therefore, the standard TK synthesis was modified to minimize the TK's molecular weight to drive the polymeric crosslink density within the cured scaffolds as high as possible. A shorter reaction time, room temperature synthesis, and maintaining a 2 : 1 stoichiometric ratio between the DOT dithiol and DMP reactants generated a TK product with a shorter chain length of *M*_n_ = 554 g mol^−1^ as determined from GPC and shown in Fig. S1.† An Ellman's assay was also used to quantify TK molecular weight by quantifying the product's molar sulfhydryl content per gram of material using a thiol standard curve (Fig. S2†). This Ellman's analysis indicated a product molecular weight of 443 g mol^−1^, agreeing well with the GPC analysis (Fig. S1†) and with the theoretical molecular weight of the 404.7 g mol^−1^ TK dimer (Table 1). When incorporated into preliminary ceramic scaffolds, the elastic moduli of the formulations increased notably when using the lower *M*_n_ TK dithiols synthesized using the optimized methods. The improved synthesis protocol for the formation of short chain TK (SCTK) dithiols was carried through the rest of the studies and is described in Fig. 1A. As guided by previously described TK polymers,^41,63,64^^1^H nuclear magnetic resonance (NMR) characterization of the SCTK in Fig. 1B shows the formation of the characteristic TK peak as expected. This NMR also indicated a lack of peaks specific to the DOT monomer (Fig. S3†), demonstrating the selective isolation of the TK product and elimination of unconjugated precursors.

**Table 1:** Molecular weight values of the SCTK product and DOT monomer as determined from theoretical structure analysis, gel permeation chromatography (GPC), and Ellman's assay

Monomer	Theoretical Mn (g mol^−1^)	GPC Mn (g mol^−1^)	Thiol content (g mol^−1^)
DOT	182.3	351 ± 12.0	220 ± 9.0
SCTK	404.7	554 ± 66.2	443 ± 10.6

**Fig. 2:**
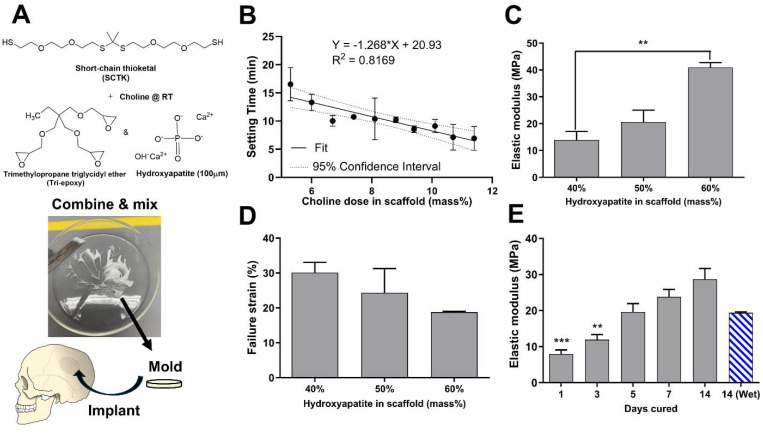
(A) Schematic for implant formation through reactive liquid mixing. (B) The tack-free time of curing TK-ceramic scaffolds was determined to have a statistically significant inverse linear relationship with the catalyst concentration (regression slope of −1.27 with 95% confidence interval of −1.76 to −0.78). Scaffolds were mechanically tested to calculate (C) elastic moduli of 40–60% HaP loaded scaffolds after five days of curing, (D) yield strain of 40–60% HaP loaded scaffolds after five days of curing, and (E) modulus values *vs.* cure time for 50% HaP loaded scaffolds (*n* = 3 samples per treatment, **p < 0.01, ***p < 0.001; indicators in E denote statistical differences for groups compared to day 14 dry samples).

To form oxidation-sensitive bone substitute materials, SCTK was combined with the epoxy terminated crosslinker trimethylolpropane triglycidyl ether (tri-epoxy), a small amount of choline to catalyze the thiol-epoxy crosslinking reaction,^65,66^ and 100 μm hydroxyapatite (HaP) crystals to form a ceramic-polymer composite once cured (Fig. 2A). Thiol-epoxy ring opening polymerization was selected here as the crosslinking chemistry for multiple purposes: TK synthesis schemes easily generate homobifunctional thiol end-groups,^41^ epoxide units rapidly form covalent linkages with thiols at room temperature,^67^ thiol-epoxy hybrid networks exhibit lower volume shrinkage than similar crosslinked materials,^68^ and multi-functional epoxides have a record of successful usage in FDA-approved biomaterials^69^ while being widely commercially available. HaP was chosen as the ceramic component for these scaffolds for numerous reasons. HaP most closely resembles the mineral phase composition found in native bone tissue compared to other calcium phosphates,^70^ mediates cellular osteoconduction,^71^ has excellent biocompatibility,^72^ and is easy to incorporate in various polymer blends to improve their strength.^73^ HaP surface properties can also drive osteoblastic differentiation during bone regeneration.^74^ Lastly, HaP has a strong record of successful clinical usage in dentistry,^75^ as coatings in total hip arthroplasty implants,^76^ and in synthetic bone grafts.^77^ Table S1† shows low sol-fraction values of less than 6% for both HaP-containing and non-Hap TK scaffolds, indicating that the molar ratio of thiols to epoxy units in the curing materials is well matched and that the HaP crystals are strongly integrated within the polymer network.

### 3.2. Bone substitute properties

PMMA based cements are notorious for possessing thermogenic properties during polymerization *in situ*,^11^ and the thiol-epoxy reaction has also demonstrated exothermic behavior in past reports.^78^ To ensure that any heat generation will be below damaging levels, the temperatures of TK ceramic scaffolds during the initial crosslinking phase following homogenization was measured (Fig. S4†). An initial gain in temperature of 1 °C was seen in the TK samples but was followed with a decrease and plateau of temperature over 15 minutes of curing. Conversely, PMMA materials achieved temperature increases of nearly 20 °C during their polymerization as shown in Fig. S4.† These results indicate that TK ceramic implants are unlikely to mediate any thermal damage to surrounding tissue unlike highly exothermic PMMA.

For successful utilization in the clinic, a defined window of working time is needed for molding and incorporation as an orthopedic bone void filler. Therefore, tack-free time of the curing TK ceramic scaffolds was measured as a function of varying catalyst concentrations to achieve a clinically-relevant 12–20 min working time.^79^ As shown in Fig. 2B, the choline concentration doped into the reactive TK/epoxy/ceramic mixture was varied from 5.3–11.4% (mass catalyst/mass of polymer, 14–32% molar equivalent) and assessed for the tack-free time when components were no longer fluid/moldable. Choline concentration at 5.3 mass % (14% mol eq.) allowed for a satisfactory working time of about 15–17 min. However, it should be noted that these materials’ curing times can be significantly shortened if desired by increasing the relative catalyst quantity as shown in Fig. 2B. A linear regression analysis was also performed to evaluate the relationship between TK scaffold setting times and the samples’ respective catalyst doses (Fig. 2B). The regression slope was quantified at −1.27 ± 0.21 (95% confidence interval), demonstrating that these two parameters possessed a statistically significant inverse relationship.

Additionally, the mechanical properties of polymer–ceramic composites can be modulated by varying the mass fraction of ceramics incorporated into final formulations. HaP crystals were incorporated into TK-epoxy mixtures at 30–70% mass fraction, loaded into 6 mm-diameter molds, allowed to cure for 5 days at room temperature, and then assessed for compressive properties as shown in Fig. 2C and D. Scaffolds formed with greater than 60% ceramic incorporation did not form cohesive products after curing, likely due to insufficient polymer binding within the microstructure. Conversely, formulations made with less than 40% ceramic exhibited inhomogeneous ceramic incorporation throughout final cured products, likely due to settling of HaP crystals before full polymer curing was achieved. Reactive polymer–HaP mixtures made with 40–60% ceramic incorporation all formed relatively homogenous and cohesive final scaffold samples. As expected, formulations with 60% HaP demonstrated the highest elastic modulus while 40% ceramic samples achieved the highest yield strain values (Fig. 2C and D). Increased ceramic content in other polymer composites has been shown to create relatively brittle materials with high modulus values but low yield strain,^24,25^ similar to pure ceramics. Given these results, scaffolds with 50% HaP loading were selected as lead candidate formulations due to their balance between ductility and strength. As shown in scanning electron microscopy (SEM) images of bisected 50% HaP TK scaffolds (Fig. S5†), these materials possessed nano-scale porosity and homogenous dispersion of HaP crystals throughout the microstructure as expected. Lastly, compressive properties of polymer–cement mixtures are known to increase over the time of curing.^43,80^Fig. 2E shows the difference of modulus values in 50% HaP scaffolds cured over two weeks, demonstrating an increase in sample rigidity over time similar to analogous composites.^43,80^ As highlighted in Fig. S6,† toughness and yield stress of curing 50% HaP scaffolds also increased over two weeks while yield strain was unchanged. Lastly, modulus values of cements incubated in PBS at 37 °C were slightly reduced compared with analogous dry samples, though the modulus differences were not statistically significant. Differences in wet *vs.* dry properties in many polymeric biomaterial scaffolds are usually attributed to the disruption of hydrogen bonds by water molecules.^81,82^ Previous TK scaffolds demonstrated negligible evidence of hydrogen bonding or differences in wet *vs.* dry mechanical properties, aligning well with these results in Fig. 2E. Overall, the ability to easily adjust ceramic quantities within these composite TK scaffolds enables simple modulation of the final implant properties.

### 3.3. Selective oxidative degradation of TK scaffolds

**Fig. 3:**
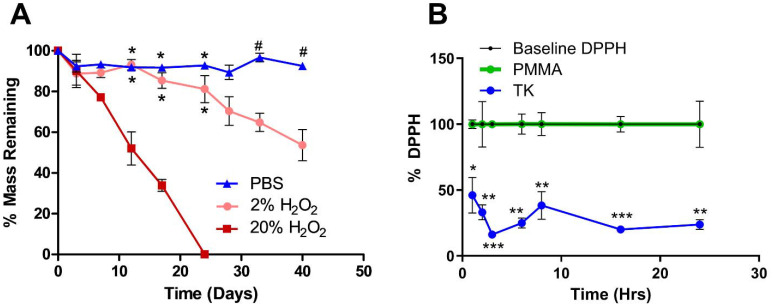
(A) TK-ceramic scaffold degradation in aqueous and oxidative media (*n* = 3 samples per treatment, *p < 0.05 comparing against 20% H_2_O_2_, ^#^*p* < 0.05 comparing against 2% H_2_O_2_). TK samples also demonstrated potent antioxidative capacity by (B) significantly reducing the activity of DPPH radicals from their baseline and when compared against PMMA (*n* = 3 samples per treatment, *p < 0.05, **p < 0.01, and ***p < 0.001 for TK *vs.* PMMA).

Current synthetic bone substitute materials are limited by poor biodegradation behavior. PMMA bone cements are designed to be biostable and last for decades *in vivo*, but their lack of resorption and remodeling by native tissue limits their osteointegration.^11^ Conversely, composite bone implants comprised of ceramics and hydrolysis-sensitive polyesters do undergo *in vivo* resorption but often degrade too quickly to achieve complete regeneration of slowly healing bone.^83^ Therefore, demonstrating the selective oxidation-mediated degradation of TK-ceramic materials was a chief goal of this work. As shown in Fig. 3A, TK-HaP scaffolds were assessed for mass loss over time by incubating samples in non-oxidative PBS or aqueous media containing 2% or 20% of the model ROS molecule hydrogen peroxide (H_2_O_2_). Scaffolds were stable in PBS over time, but when incubated in the H_2_O_2_-containing media, were significantly degraded and broke down more quickly when exposed to higher concentrations of ROS (Fig. 3A). Additionally, TK scaffolds incubated in PBS underwent minimal swelling over 7 days while samples treated with 20% H_2_O_2_ media over the same period displayed a four-fold increase in swelling (Table S2†). These results align well with past examples of bulk TK biomaterials that are inert to hydrolysis but undergo dose-dependent degradation in oxidative environments.^41,43,46^ This material breakdown by cell-generated molecules will also ideally allow for these TK-ceramic scaffolds to be fully cleared from the body over time when implanted *in vivo.*

Additionally, the antioxidant capacity of these scaffolds was evaluated since past TK biomaterial implants have demonstrated potent scavenging of damaging radicals and corresponding increases in tissue regeneration.^58,84^ Scaffolds were incubated with the free radical-containing molecule 1,1-diphenyl-2-picrylhydrazil (DPPH) to assess DPPH inactivation as indicated by quantitative absorbance measures of color change.^55,56^ When compared against baseline DPPH signal, a significant decrease in DPPH activity was observed with incubated TK scaffolds whereas the DPPH signal was nearly unchanged with PMMA materials (Fig. 3B). These results confirm the antioxidant character of TK biomaterials and limited antioxidant capacity of commercial bone implants. Additionally, TK-ceramic scaffolds with differing levels of HaP incorporation (40, 50, or 60% mass fraction) were similarly incubated with DPPH for 24 h as shown in Fig. S7.† All TK-ceramic formulations demonstrated a significant reduction in DPPH signal as expected, but samples with higher amounts of ceramic incorporation had less radical scavenging likely because of a reduced amount of antioxidant TK material in the samples (Fig. S7†). These results further confirm that the TK component is the main antioxidative mediator in these scaffolds. From recent reviews, biomaterials with antioxidant capacity have demonstrated improved healing outcomes in skin wounds^37,85^ and bone defects^40,86,87^ due to a decrease in oxidative stress within the implantation site. A particular strength of TK-based implants is their ability to naturally detoxify ROS without needing to add exogenous antioxidant molecules.^46,88^ This innate antioxidant capacity further demonstrates the potential of TK-based implants for repairing bone defects as alternatives to current PMMA chemistries.

### 3.4. *In vitro* cytotoxicity of TK-ceramic scaffolds

**Fig. 4:**
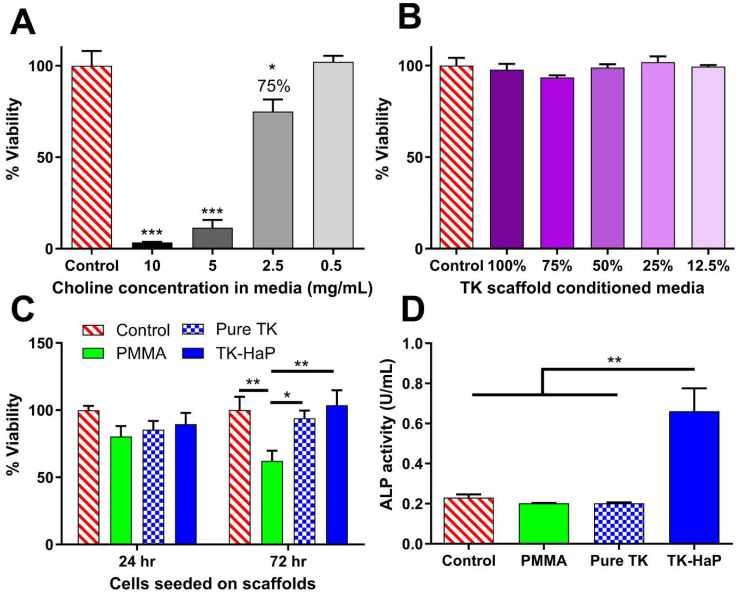
(A) Cytotoxicity analysis of increasing concentrations of choline-doped cell media incubated with MC3T3-E1 cells. (B) Elution cytotoxicity testing with MC3T3-E1 cells and dilutions of media conditioned with TK-ceramic scaffolds following ISO 10993-5 standards. (C) Cytotoxicity testing with MC3T3-E1 seeded directly on PMMA, pure TK, and TK-HaP scaffolds for 24 and 72 h. (D) Osteogenic activitiy of MC3T3-E1 cells incubated for 7 days with PMMA, pure TK, and TK-HaP scaffolds in osteogenic media (*n* = 3 samples per treatment, *p < 0.05, **p < 0.01, ***p < 0.001).

Acute cytotoxic concentrations of choline have not previously been reported. Due to its use within these scaffolds to catalyze thiol-epoxide crosslinking, cytotoxicity analysis of choline in cell media was performed with MC3T3-E1 pre-osteoblast cells for a 24 h treatment time as shown in Fig. 4A. A 50% lethal concentration (LC50) of 3.5 mg mL^−1^ was determined for choline under these conditions. A concentration of 2.5 mg mL^−1^ generated a slight decrease in cellular viability (Fig. 4A) and roughly corresponded with a 5.3 mass % catalyst dose (14% mol eq.) that achieved a scaffold working time of 16 min (Fig. 2B) in hardening TK-epoxy scaffolds. This catalyst level was therefore determined as appropriate for use in these curing materials.

Using protocols adapted from ISO 10993-5, the toxicity of crosslinked TK-ceramic scaffolds was also determined using an elution-based toxicity test. 50% HaP TK samples were fabricated, cured for 5 days at room temperature, and incubated in cell media for 24 h before harvesting the conditioned media. This media was then diluted, administered to MC3T3-E1 cells for 24 h, and finally assessed for cytotoxicity using a CellTiter-Glo assay. As demonstrated in Fig. 4B, no statistically significant differences in toxicity were observed between cells treated with control media or media conditioned with scaffold eluate at any dilution. To completely capture cellular compatibility with TK scaffolds, MC3T3-E1 cells were also seeded directly onto TK-HaP composites, TK polymer scaffolds without HaP, and PMMA control materials. Cell viability was quantified using a CellTiter-Glo assay after 24 h and 72 h following initial cell seeding. As shown in Fig. 4C, all scaffold formulations hosted similar numbers of viable cells after 24 h but did show a divergence at 72 h with the TK scaffolds significantly out-performing the PMMA formulation. The cytocompatibility of TK scaffolds was expected given the limited *in vitro* toxicity seen in other TK biomaterial systems^33,41,43^ and the relatively low dose of choline catalyst used to form these samples (Fig. 4A).

To highlight the osteogenic capability of TK-ceramic scaffolds, alkaline phosphatase (ALP) levels were quantified from differentiating MC3T3-E1 pre-osteoblasts that were incubated with TK, TK-HaP, or PMMA scaffolds. As shown in Fig. 4D, cells incubated with lead candidate TK-HaP composite materials demonstrated significantly increased ALP activity levels compared to PMMA or the non-ceramic pure TK samples. Given the previous use of HaP as an osteoconductive additive in orthopedic implants,^70,71^ it is relatively unsurprising that HaP-loaded TK composite materials increased the osteogenesis of treated cells. Nevertheless, these findings further established the TK-HaP (50% ceramic loading) formulation as lead candidate materials that would be carried forward to *in vivo* testing.

### 3.5. *In vivo* regeneration of critically-sized bone defects following TK and PMMA scaffold implantation

Sprague Dawley rats (*n* = 4 per group) were utilized in a critically-sized cranial defect model to assess the osteogenic properties of TK and PMMA implants. Using established techniques,^62^ 8 mm-diameter defects were surgically created by trephination in the calvarial bone of rats before implanting the respective scaffold formulations. Calvarial defects of 8 mm are generally recognized as “critically-sized” in that the animals cannot completely heal this large of a bone injury over their natural lifetime.^62^ Moreover, this non-healing bone defect model has been widely implemented throughout the literature while investigating novel orthopedic implant materials.^89^ Many investigators have produced negative control data using this rodent model by leaving the 8 mm defects empty and showing conclusive evidence of non-regeneration.^90,91^ Consequently, these prior literature examples of minimally-healing calvarial defects were deemed appropriate for providing historical controls for this *in vivo* study. To therefore reduce the number of animals used in this experiment following the principles of the 3Rs for animal research (Replacement, Refinement, and Reduction),^92^ a negative control group with empty calvarial defects that reproduced well-established findings was not included. Conversely, PMMA was selected as the “standard treatment” control material for these *in vivo* studies due to its long history of use in clinical orthopedic procedures,^11^ FDA approval as a bone cement,^93^ wide employment as a synthetic bone substitute material,^94^ and its categorization as a synthetic polymer to best compare against the synthetic TK polymer implants. While other implant systems have shown robust bone regeneration outcomes in this calvarial defect model using exogenous growth factors^95–97^ or stem cells,^98–100^ the goal of this experiment was to establish baseline levels of new bone growth promoted by the TK materials. As the polymeric implant most commonly used in bone reconstruction procedures,^11^ PMMA was deemed the most appropriate control material to compare against these newly-formulated polymeric TK composites.

**Fig. 5:**
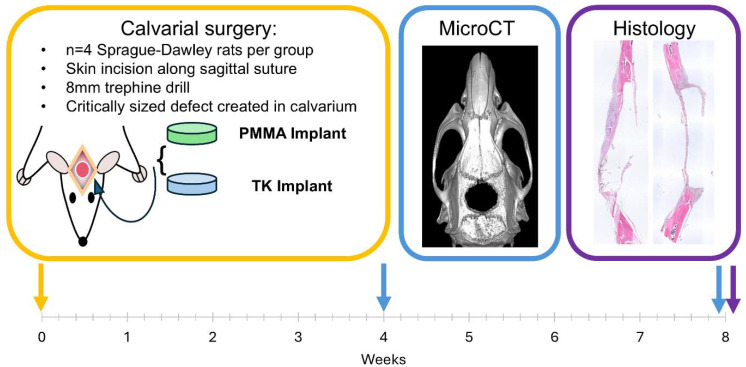
Calvarial surgery implant study timeline summary. These studies were conducted for 8 weeks in total; live animal microCT imaging was performed at week 4 while post-mortem CT and tissue collection for histology were performed at week 8.

**Fig. 6:**
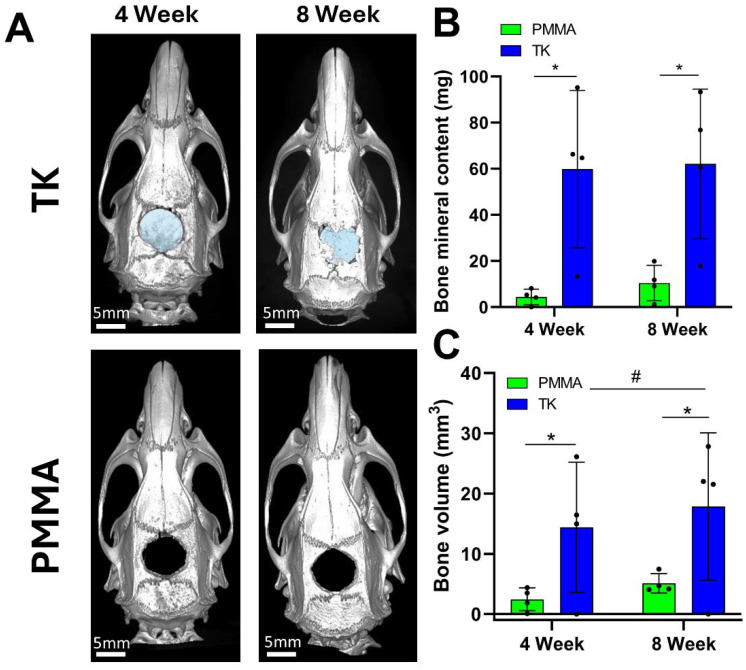
(A) MicroCT visualization of bone growth around TK-HaP and PMMA scaffolds implanted in critically-sized 8 mm calvarial defects (4 and 8 weeks post-operation). Blue colored spaces indicate TK implants. When substracting out the mineralized TK scaffolds, quantification of (B) bone mineral content and (C) bone volumes within the 8 mm defects demonstrates significant bone growth in the TK-treated animals (*n* = 4 samples per treatment, *p < 0.05 for TK *vs.* PMMA within same timepoint, ^#^*p* < 0.05 comparing TK and PMMA treatments from week 4 to week 8).

The experimental workflow for these rodent studies is outlined in Fig. 5: after implanting materials into the surgical defects at week 0, microcomputed tomography (microCT) was used to quantify bone regeneration at 4 and 8-weeks post operation. MicroCT is considered a gold-standard methodology for assessing *in vivo* tissue calcification and bone regeneration since it can quantify radiopaque calcium deposits throughout an entire tissue volume.^91^ This technique offers distinct advantages over biochemical analytical methods or immunohistopathology which rely on harvesting small sections of tissue, lack spatial resolution of volumetric bone growth, and can suffer from sampling bias. Representative microCT images of the regenerating calvarial defects are shown in Fig. 6A; it should be noted that the TK composite implants can be visualized in the microCT images (highlighted with false-color blue shading) since they contain radiopaque HaP ceramic particles. As shown in Fig. S8,† TK-ceramic scaffolds and nascent bone had very similar CT signal values that could not be easily separated by thresholding in the Microview analysis software. The clinical control PMMA implants, conversely, did not contain ceramic content and were therefore more easily thresholded out from the bone tissue's CT signal. Critically, the CT images in Fig. 6A show bony bridge formation around TK scaffolds at both time points while PMMA implants induced little to no bone regeneration in the defects.

Additionally, quantified bone volume (BV) and bone mineral content (BMC) values specifically collected within the defect margins demonstrated significant differences between PMMA and TK implants (Fig. 6B and C). Defects with TK scaffolds possessed significantly higher BV and BMC at 4 and 8 weeks post operation compared to PMMA. Moreover, TK samples facilitated significantly increased bone volumes within the defects from 4 to 8 weeks, indicating the on-going bone regeneration spurred by the TK materials. These bone metrics were quantified by subtracting out the signal from the TK implant to isolate the BV and BMC values of new bone tissue within the defects. The BV and BMC values for a naive TK-ceramic scaffold were determined from a microCT scan of a TK implant within a day 0 calvarial defect in a cadaver rat (Fig. S9†). Though difficult to ascertain, it is also possible that this methodology undercounted new bone growth since TK implants would likely have decreased CT signal values over time as they were degraded. Nonetheless, the microCT bone visualization and quantification outcomes both demonstrate significant increases in bone re-growth for animals treated with TK-ceramic scaffolds compared to the PMMA clinical controls.

**Fig. 7:**
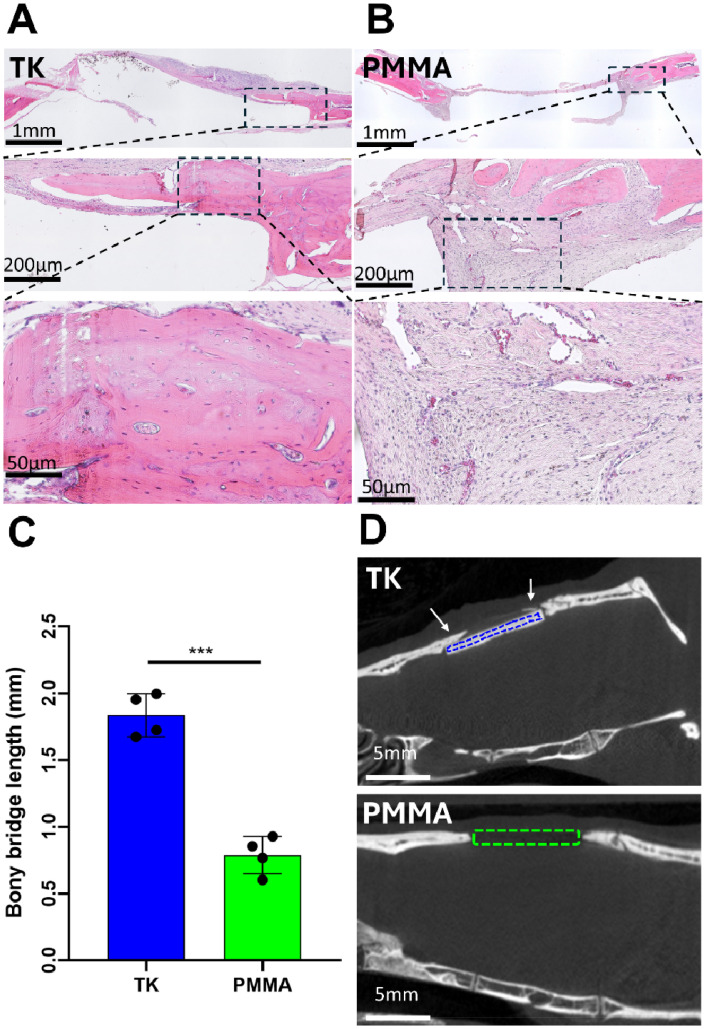
2D visualizations of bone regeneration in calvarial defects following TK or PMMA scaffold treatment. (A) H&E-stained histology sections of 8-week calvarial defects implanted with TK scaffolds show increased bone growth around the TK materials compared with (B) fibrous tissue formation surrounding PMMA samples. Both TK and PMMA implants were removed during histological processing so only the surrounding tissue is evident. (C) Quantification of bony bridge length from H&E histology in the calvarial defects treated with TK and PMMA implants. (D) MicroCT 2D cross sections of TK and PMMA implants (indicated by dashed outlines) in 8 mm calvarial defects after 8 weeks of implantation. White arrows indicate bony bridge formation across the defect site (***p < 0.001).

To further visualize tissue interactions around the TK and PMMA implants, animals were sacrificed at week 8 to collect tissue explants from the calvarial defects and processed *via* hematoxylin & eosin (H&E) histology as shown in representative images in Fig. 7A and B. As evidenced in these images, calvarial defects treated with TK-ceramic scaffolds showed significant bony bridge formation though not full defect closure over 8 weeks (Fig. 7A). Conversely, PMMA samples demonstrated negligible new bone growth into the defect site (Fig. 7B). When quantifying bony bridge length in defects respectively treated with TK or PMMA implants from histology, the TK scaffolds demonstrated twice the bone growth of PMMA analogues (Fig. 7C). The bone–implant interfaces also differed between the TK and PMMA materials. Tissue surrounding TK implants consisted of mineralized and regenerating bone with a woven morphology, whereas PMMA samples featured almost exclusively fibrotic soft tissue enveloping them as highlighted in Fig. 7B. Slices of 2D microCT images from 8-week defects with respective TK or PMMA implants further confirm these findings by showing new mineralized bone tissue exclusively developing in TK-treated animals (Fig. 7D). The baseline tissue morphology of a day 0 calvarial defect is also shown in Fig. S10,† demonstrating the initial microstructure of native bone before it has been influenced by exogenous materials. In short, the differential 8-week tissue response between bone-promoting TK samples and fibrosis-inducing PMMA implants highlight the regenerative potential of these new biomaterials.

## 4. Conclusions

Biomaterials featuring responsive TK chemistries represent a promising new class of medical implant due to their selective degradation by oxidative free radicals and ability to facilitate cell-driven material degradation. Here, easily-synthesized TK precursors were used to develop ROS-responsive, *in situ*-curing synthetic bone substitutes that form *via* efficient thiol-epoxy ring opening polymerization. These scaffolds were also loaded with HaP ceramics to enhance their mechanical loading and osteogenic capacity, and material properties such as curing time and compressive strength were respectively tuned by varying the catalyst dose or levels of ceramic incorporation. TK scaffolds demonstrated long term stability in aqueous conditions but were selectively degraded in ROS-rich environments in a dose-dependent manner. Using a free-radical scavenging assay, the antioxidative capacity of these materials was also demonstrated and shown to be dependent on the quantity of TK in the sample. Additionally, TK-ceramic scaffolds were not cytotoxic in elution or direct contact cell viability assays and elicited potent cellular osteogenesis *in vitro*. Lastly, *in vivo* evaluation of these materials in a critically-sized bone defect rodent model demonstrated a significant increase in bone regeneration fostered by TK scaffolds compared against clinical control PMMA samples. TK implants induced greater bone development and bony bridge formation compared to PMMA analogues as confirmed *via* microCT and histological analyses. Together, these results highlight the utility of synthetic, oxidation-responsive bone substitute materials and demonstrate their potential as regenerative therapies for large-scale bone defects.

## Author contributions

R.L.D.: conceptualization, data curation, formal analysis, investigation, methodology, project administration, resources, supervision, validation, visualization, writing – original draft, writing – review & editing. A.A.: resources, investigation. B.E.H.: investigation. A.E.A.: investigation. D.W.M.: investigation. K.A.B.: investigation. J.R.M.: conceptualization, funding acquisition, project administration, resources, supervision, writing – review & editing.

## Conflicts of interest

Authors declare no conflicts of interest in this publication.

## Supplementary Material

BM-013-D4BM01345J-s001

## Data Availability

Data pertaining to any portion of the publication are available upon request. The data supporting this article have been included as part of the ESI.† Raw data collected and used in the presentation of the work is available upon request.
