# Supplementary material for: Oxidation-responsive, settable bone substitute composites for regenerating critically-sized bone defects
Source: Biomater Sci. 2025 Feb 21;13(8):1975–92. doi: 10.1039/d4bm01345j (PMC11877281; doi:10.1039/d4bm01345j)
Supplement: BM-013-D4BM01345J-s001 [file BM-013-D4BM01345J-s001.pdf]

Supplementary Information (SI) for Biomaterials Science.
This journal is © The Royal Society of Chemistry 2025

Oxidation-responsive, settable bone substitute composites for regenerating critically-sized bone defects

[Reinaldo L. Dos Santos, Ardeena Ahmed, Brooke E. Hunn, Adolphus E. Addison, Dylan W. Marques, Karina A. Bruce, and John R. Martin]

Supplemental Tables, Figures, and Methods

Table S1 – Sol-fraction of TK ceramic scaffolds

	Sol-fraction (%)	
n=5	HaP loaded	No HaP
Average	3.6	5.0
SD	0.6	0.6

Table S2 - Swelling ratio values of TK-ceramic scaffolds

Day 1: PBS incubation	Day 7: PBS incubation	Day 7: 20% H_2O_2 incubation
8.59 ± 0.5	10.9 ± 1.3	41.6 ± 6.0

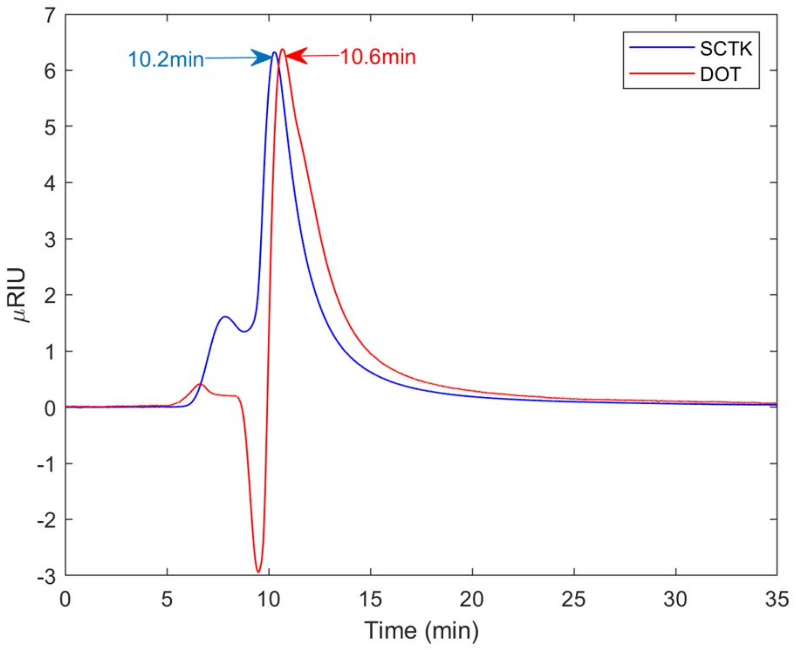

Figure S1 –Gel permeation chromatogram of SCTK and precursor monomer DOT showing a molecular weight increase for the polymerized SCTK product

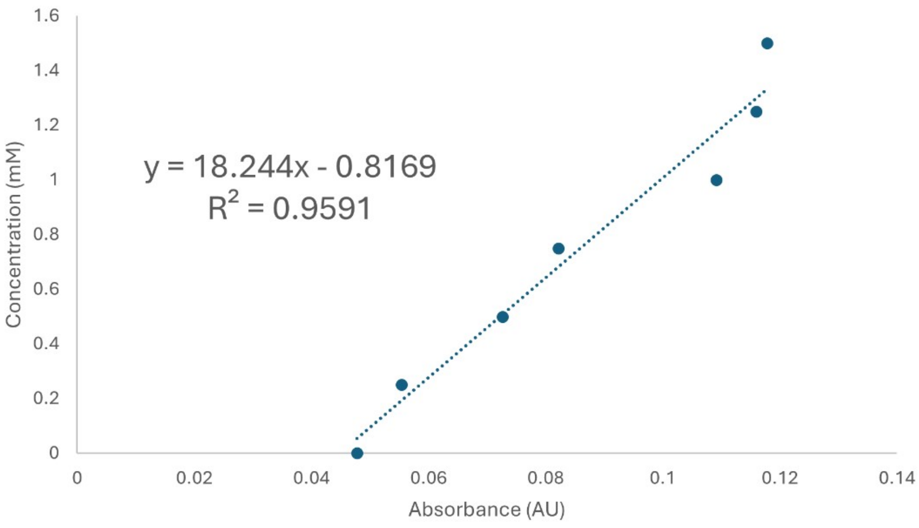

Figure S2 – Standard curve graph of Ellman's assay used to estimate thiol content of SCTK monomer

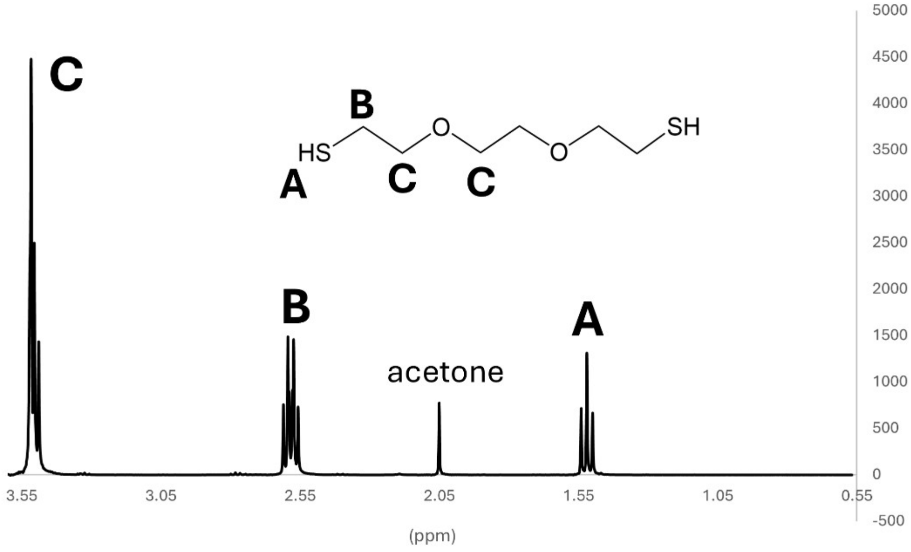

Figure S3 – ^{1}H-NMR of the initial dithiol monomer 3,6-dioxa-1,8-octanedithiol (DOT) used to form SCTK

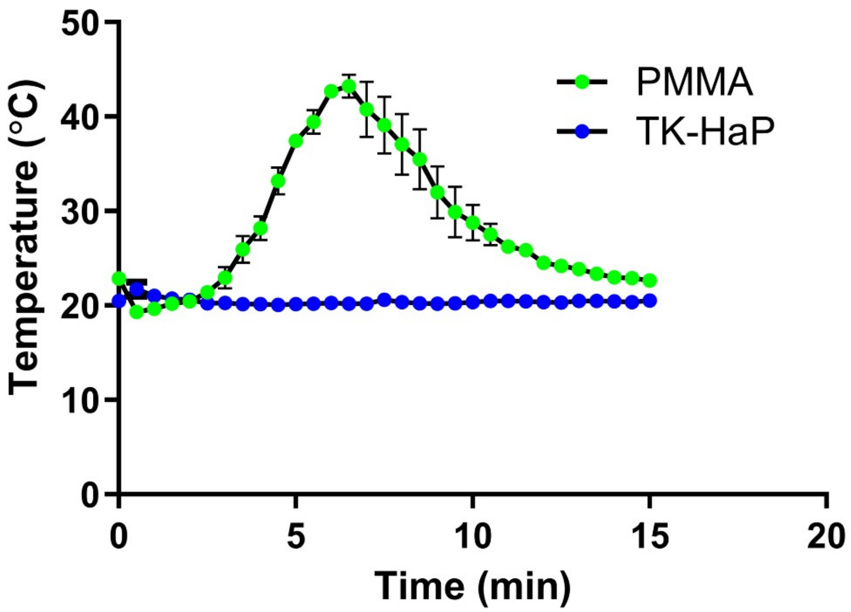

Figure S4 – Temperature readings of TK and PMMA materials during hardening.

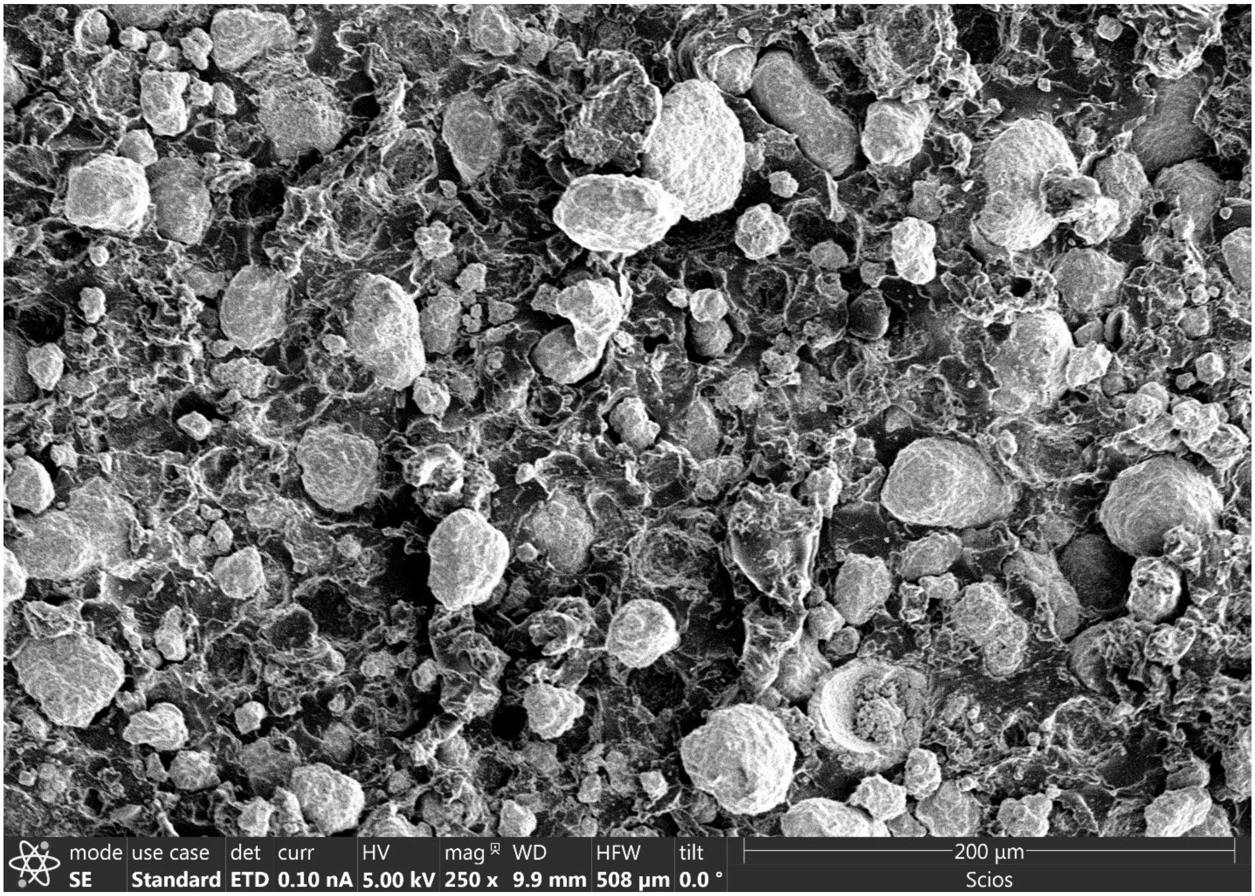

Figure S5 – Scanning electron microscopy (SEM) images of bisected 50% HaP TK scaffolds

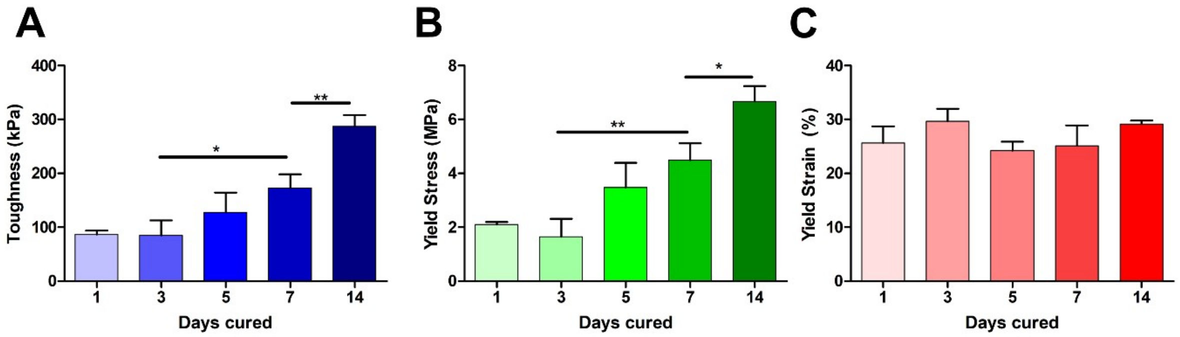

Figure S6 – Compressive properties of 50% HaP TK scaffolds , including (**A**) toughness (calculated from area under the stress-strain curves, (**B**) yield stress, and (**C**) yield strain (n=3 samples per treatment time *p<0.05, **p<0.01).

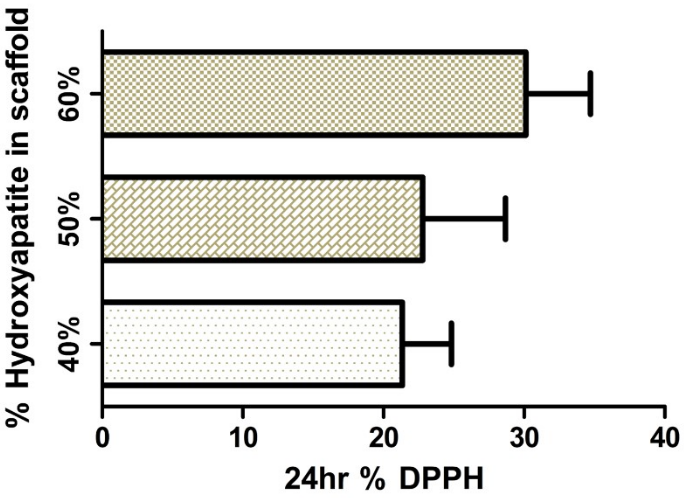

Figure S7 – DPPH antioxidant activity of TK scaffolds with variable HaP loading.

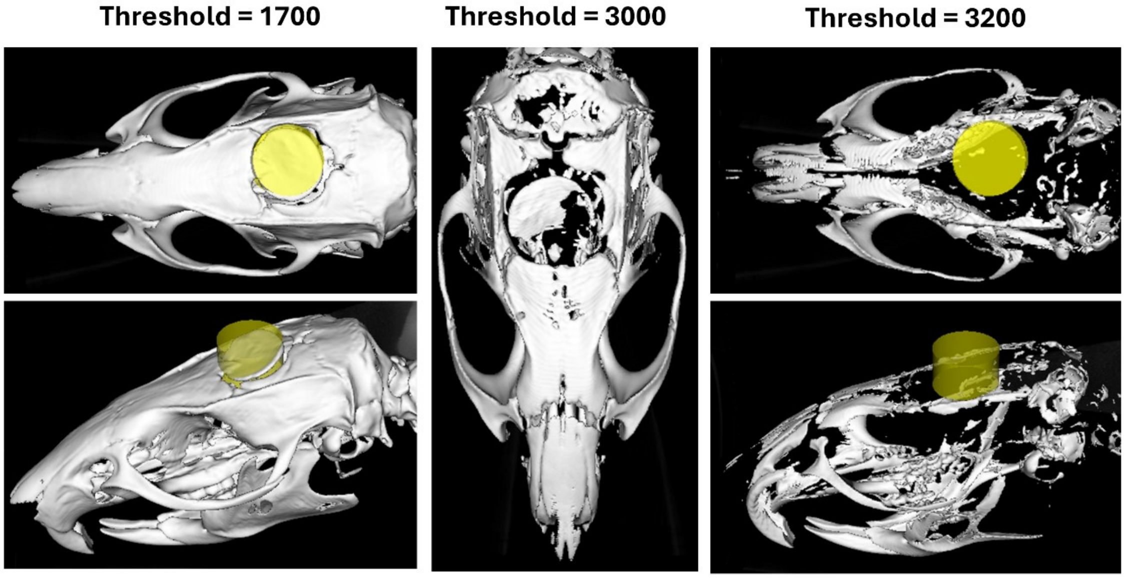

Figure S8 – Various isosurface visualizations of rat skulls at different thresholds from microCT readings. Various thresholds were used to illustrate how bone analysis was performed without neglecting newly forming nascent bone.

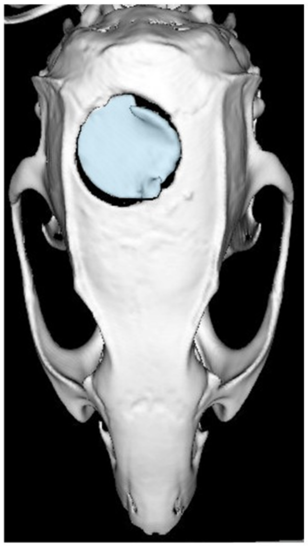

Figure S9 – MicroCT of a day 0 calvarial defect with an implanted TK scaffold (light blue shading). This scan was used for quantification of HaP mineral phase contribution to bone volume and bone mineral content during bone analysis in Microview.

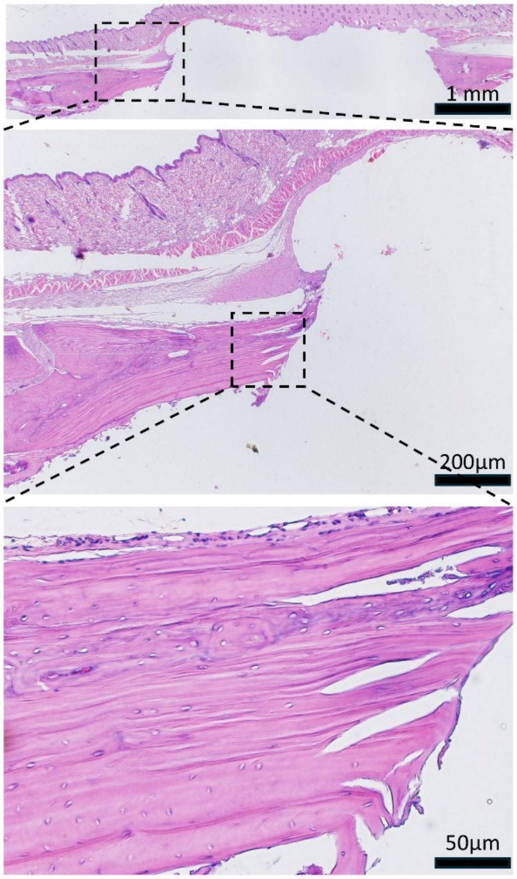

Figure S10 – Day 0 H&E stained calvarial defects

Microcomputed tomography bone analysis

Bone volume and bone mineral content were determined using Microview open source software and the included bone analysis tool. This allowed for quantitative calculation of bone regeneration between TK and PMMA implanted scaffolds at 4 and 8 weeks post operation. To perform this analysis, threshold values corresponding to Houndsfield units (HU) of bone and water content in soft tissue were first inputted to the analysis package. However, calcium phosphate in hydroxyapatite (HaP) is similar to the mineral phase of bone and therefore its contributions to bone volume (BV) and bone mineral content (BMC) were excluded from the bone analysis.

Threshold values for bone at 3200 eliminated the signal associated with TK scaffolds. However, this also eliminated much of the signal from less dense bone around the implant site and from nascent bone being generated around the implant. Isosurfaces at increasing threshold values were generated to illustrate the influence of thresholding on collected bone signal (Figure S8). Therefore, bone analysis determining BV and BMC at a threshold of 3200 did not accurately represent what was observed throughout our histological analysis of these tissues. To achieve an accurate assessment of regenerated bone, day 0 implanted samples were scanned to determine baseline BV and BMC values from non-integrated TK scaffolds at our chosen threshold of 1700 (Figure S9). BV and BMC values collected from microCT scans of TK implants at 4 and 8-week were adjusted to subtract out the TK-only signal collected at day 0. This was done so that BV and BMC values of newly generated bone could be more accurately determined. The final numbers in TK scaffold scans are reported as BV and BMC values subtracted by BV and BMC from day 0 samples. This was not necessary to do with PMMA scaffolds since PMMA is radiopaque compared to HaP-containing TK scaffolds.
